# How to rule out non-neoplastic hypercortisolemia (previously known as pseudo-cushing)

**DOI:** 10.1007/s11102-022-01222-2

**Published:** 2022-05-26

**Authors:** Carla Scaroni, Alessandro Mondin, Filippo Ceccato

**Affiliations:** 1 Department of Medicine (DIMED) and Rare Diseases (DIMAR), University of Padova, Padova, Italy; 2 Endocrinology Unit, Hospital-University of Padova, Padova, Italy; 3 Endocrinology Unit, Department of Medicine DIMED and of Rare Diseases DIMAR, Via Ospedale Civile, 105-35128 Padova, Italy

**Keywords:** Cushing’s syndrome, pseudo-Cushing’s syndrome, CRH test, Desmopressin test, dexamethasone-CRH test

The term non-neoplastic hypercortisolism (NNH) is used frequently nowadays, interchangeable with the pseudo-Cushing (pCS) state, in order to differentiate the functional non-neoplastic hypercortisolism that mirrors the neoplastic activation of the HPA axis, termed endogenous Cushing’s syndrome (CS). Diagnosis of neoplastic CS is often complex, especially when modest cortisol excess is present. Indeed, functional activation of the hypothalamic-pituitary-adrenal (HPA) axis can be caused by a variety of illnesses/conditions (e.g., psychiatric disorders, alcoholism, obesity, or polycystic ovary syndrome) [1]. Although some clinical features (such as bruisability, facial plethora, proximal myopathy, and large purple cutaneous striae) are highly specific for CS, they are not sensitive. Furthermore, NNH/pCS-related conditions may have clinical characteristics that overlap with CS, making the differential diagnosis very challenging [2].

Before ruling out NNH/pCS with second-line testing the possibility of a false-positive result from first-line testing for hypercortisolism should be considered. The low specificity of immunoassays may cause false-positive results from interfering circulating steroids (mainly cortisone) in late-night salivary cortisol and urinary-free cortisol. The use of estrogen-progesterone contraceptives or CYP3A4 inducing drugs may cause false-positive results at low-dose dexamethasone suppression test (LDDST). Special attention to the pre-analytical phase (clear and written instructions for sample collection, withdrawal of interfering drugs), use of mass spectrometry assays, and dexamethasone (dex) measurement may be useful tools to avoid false-positive results. In addition, physicians should also select the most appropriate test for the considered patient: avoid measuring circadian rhythm in shift workers, urinary cortisol in case of impaired renal function, and so on [3].

Several second-line tests have been proposed to distinguish NNH/pCS from neoplastic CS (Table 1), but there is still no agreement on the gold standard one among them. The combined dex-Corticotropin Releasing Hormone (dex-CRH) test assumes that only CS patients will sustain a cortisol response to CRH stimulation following dex suppression, allowing NNH/pCS and CS to be distinguished by specified thresholds. Proposed by Yanovski et al. in 1993, the combination of LDDST and CRH test for in-patients (the cohort of pCS subjects mainly presenting with affective disorders) with a cut-off for cortisol level at 15’ after CRH stimulation over 38 nmol/l, reported 100% sensitivity and specificity [4]. Despite stricter cortisol cut-offs and consideration of stimulated-ACTH levels, other studies did not corroborate this high diagnostic accuracy. Different study protocols (number and time of dex doses, ovine vs human CRH at fixed/weight-adjusted doses), different accuracy of cortisol assays for low levels, concomitant medications, even without measurement of serum dex, and differences in the enrolled population (number of cases, severity of hypercortisolism, subtypes of CS and NNH/pCS) can all contribute to different results [5]. Other tests, such as LDDST [6], midnight serum cortisol [7], and the desmopressin (DDAVP) test [8] indicated greater NNH/pCS detection accuracy. CRH test (without dex suppression) is useful in the differential diagnosis of ACTH-dependent CS; nonetheless, it performed poorly in Yanowski’s study in terms of discrimination between NNH/pCS [4]. Arnaldi et al gave it new life by using a bimodal cut-off and generating promising results [9, 10] that would allow physicians to confirm CS and learn more about the cause of ACTH excess with a single test. Further studies are however needed to confirm these findings.

**Table 1:** Second line tests in the differential diagnosis of CS vs. pCS. Δ = Delta, dex = dexamethasone, CRH = Corticotropin Realising Hormone, DDAVP = Desmopressin, CS = Cushing’s Syndrome, CD = Cushing’s Disease, pCS = pseudo-Cushing’s syndrome, ACS = Adrenal Cushing’s Syndrome, EAS = Ectopic ACTH Secretion, Se = Sensitivity, Sp = Specificity. *Paediatric patients

Study	Test	CS	pCS / controls	Cut-off	Se (%)	Sp (%)
Yanovski JA, 1993	Dex-CRH	CD 35, ACS 2, EAS 2	pCS 19	Cortisol at 15′: 38 nmol/L (1.4 µg/dL)	100	100
Martin NM, 2006	Dex-CRH	CD 8, ACS 4	pCS 3, controls 16	Cortisol at 15′: 50 nmol/L (1.8 µg/dL)	100	88
Gatta B, 2007	Dex-CRH	CD 17 (mild)	pCS 14	Cortisol at 15′: 110 nmol/L (4 µg/dL)	100	86
				ACTH at 15′: 3.5 pmol/L (16 pg/mL)	100	85
Erickson D, 2007	Dex-CRH	CD 21	pCS 30	Cortisol at 15′: 70 nmol/L (2.5 µg/dL)	90	90
				ACTH at 15′: 5.9 pmol/L (27 pg/mL)	95	97
Pecori-Giraldi F, 2007	Dex-CRH	CD 29, ACS 3	pCS 23	Cortisol at 15’: 75 nmol/L (2.7 µg/dL)	100	82
Reimondo G, 2008	Dex-CRH	CD 13, ACS 3	pCS 15	Cortisol at 15′: 44 nmol/L (1.6 µg/dL)	93.7	93.3
Valassi E, 2009	Dex-CRH	CD 60	pCS 41	Cortisol at 15′: 38 nmol/L (1.4 µg/dL)	86.3	84.7
Alwani RA, 2014	Dex-CRH	CD 53	pCS 20	Cortisol at 15′: 87 nmol/L (3.2 µg/dL)	94	100
Batista D, 2008*	Dex-CRH	CS 11	pCS 11 (Obese)	Cortisol at 15′: 88 nmol/L (3.2 µg/dL)	91	95
Yanovski JA, 1993	CRH	CD 35, ACS 2, EAS 2	pCS 19	Sum of post-CRH cortisol levels > 3450 nmol/L (125 µg/dL)	64	100
Arnaldi G, 2009	CRH	CD 51, EAS 7	pCS 26, controls 31	Basal serum cortisol > 331 nmol/L (12 µg/dL) and ACTH peak > 12 pmol/L (54 pg/mL)	91.3	98.2
Tirabassi G, 2011	CRH	CD 30	pCS 18, controls 12	Basal serum cortisol > 331 nmol/L (12 µg/dL) and ACTH peak > 12 pmol/L (54 pg/mL)	96.6	100
Malerbi DA, 1996	DDAVP	CD 14	pCS 11	Δ-Cortisol ≥ 4 times intra-assay variation coefficient	100	64
Moro M, 2000	DDAVP	CD 76	pCS 30, controls 67	Δ-ACTH ≥ 6 pmol/L 0’ – 30′ (27.2 pg/mL)	86.8	90.7
		CD 20 (mild)	pCS 30		90	96.7
Pecori-Giraldi F, 2007	DDAVP	CD 27	pCS 21	Δ-ACTH ≥ 6 pmol/L 0’ – 30′ (27.2 pg/mL)	81.5	90
Tirabassi G, 2010	DDAVP	CD 52	pCS 28, controls 31	Δ-ACTH > 6 pmol/L 0’ – 30′ (27.2 pg/mL)	75	89.8
				Basal serum cortisol > 331 nmol/L (12 µg/dL) and Δ-ACTH > 4 pmol/L (18 pg/mL)	90.3	91.5
Tirabassi G, 2011	DDAVP	CD 30	pCS 18, controls 12	Basal serum cortisol > 331 nmol/L (12 µg/dL) and Δ-ACTH > 4 pmol/L (18 pg/mL)	96.6	100
Rollin G, 2015	DDAVP	CD 68	pCS 56	Peak ACTH of 15.8 pmol/L (36.8 pg/mL)	90.8	94.6
				Δ-ACTH ≥ 8.1 pmol/L 15′–30′	88	96.4
Araya V, 2017	Dex-DDAVP	CD 36	pCS 9, controls 7	Δ-Cortisol ≥ 166 nmol/L (6 µg/dL)	96.9	93.7

ACTH-secreting adenomas may aberrantly express vasopressin receptor 2 (VR-2). As a result, the synthetic analog DDAVP could only elicit ACTH response in Cushing’s Disease (CD) patients, because the normal pituitary expresses VR-3, which has a low DDAVP affinity [11]. A rise in ACTH levels above 6 pmol/L has been proposed to differentiate CD from NNH/pCS, achieving the best result in patients with mild hypercortisolism [12]. An alternative bimodal cut-off has been proposed to boost its accuracy [13]; both approaches demonstrated acceptable results in a larger retrospective cohort [14]. This test is simpler and less expensive than dex-CRH, and it is available in the US and other countries. In regions where CRH is not available, Araya et al. recommended a dex-DDAVP test as an alternative, employing cortisol as a less expensive readout [15]. In a direct comparison of dex-CRH and DDAVP, Pecori Giraldi et al. found that the latter performed better in confirming CD. As a result, a step-by-step approach involving a sensitive first-line test (e.g., LDDST) followed by DDAVP may be the most effective. [8]. Martin et al., on the other hand, pointed out that increasing the cortisol cut-off in dex-CRH in the same series could boost specificity, diminishing the claimed advantage [16]. Tirabassi et al. also directly compared the accuracy of the DDAVP and CRH tests, finding excellent and similar results. The two tests also had a high level of diagnostic agreement, resulting in no CS misdiagnosis [10].

No single test among those proposed seems to guarantee a perfect distinction between CS and NNH/pCS patients. Furthermore, the wide variation in the population recruited, the NNH/pCS definition criteria, and the cut-offs used in different studies make it difficult to define a gold standard in this intricate differential diagnosis. To summarize, ruling out NNH/pCS-related disorders is required in cases of moderate hypercortisolism, equivocal clinical presentation, or suspected non-neoplastic hypercortisolism. According to the recent Pituitary Society consensus report, the patient’s clinical history and the duration of symptoms are of utmost importance in the differential diagnosis of NNH/pCS [17]. The specific treatment of the underlying condition that led to non-neoplastic hypercortisolism can achieve the recovery of HPA-axis function: clinical assessments and first-line testing should be repeated 3–6 months after baseline [1, 3, 17]. Second-line dynamic tests can be performed in referral centers with expertise in the differential diagnosis of CS and knowledge of their cut-offs. To rule out a CD diagnosis in a doubtful circumstance, a skilled utilization of dynamic diagnostics along with frequent clinical and hormonal surveillance is required [2, 17].

## Data Availability

All data generated or analyzed during this study are included in this published article.

## References

[1] Findling JW, Raff H, DIAGNOSIS OF ENDOCRINE DISEASE (2017). Differentiation of pathologic/neoplastic hypercortisolism (Cushing’s syndrome) from physiologic/non-neoplastic hypercortisolism (formerly known as pseudo-Cushing’s syndrome). Eur J Endocrinol.

[2] Scaroni C, Albiger NM, Palmieri S (2020). Approach to patients with pseudo-Cushing’s states. Endocr Connect.

[3] Petersenn S (2021). Biochemical diagnosis of Cushing’s disease: Screening and confirmatory testing. Best Pract Res Clin Endocrinol Metab.

[4] Yanovski JA, Cutler GB, Chrousos GP, Nieman LK (1993). Corticotropin-releasing hormone stimulation following low-dose dexamethasone administration. A new test to distinguish Cushing’s syndrome from pseudo-Cushing’s states. JAMA.

[5] Nieman L, Editorial (2007). The dexamethasone-suppressed corticotropin-releasing hormone test for the diagnosis of Cushing’s syndrome: what have we learned in 14 years?. J Clin Endocrinol Metab.

[6] Martin NM, Dhillo WS, Banerjee A (2006). Comparison of the dexamethasone-suppressed corticotropin-releasing hormone test and low-dose dexamethasone suppression test in the diagnosis of Cushing’s syndrome. J Clin Endocrinol Metab.

[7] Gatta B, Chabre O, Cortet C (2007). Re-evaluation of the combined dexamethasone suppression-corticotropin-releasing hormone test for differentiation of mild Cushing’s disease from pseudo-Cushing’s syndrome. J Clin Endocrinol Metab.

[8] Pecori Giraldi F, Pivonello R, Ambrogio AG et al (2007) Sep;67(3):477] The dexamethasone-suppressed corticotropin-releasing hormone stimulation test and the desmopressin test to distinguish Cushing’s syndrome from pseudo-Cushing’s states [published correction appears in Clin Endocrinol (Oxf). Clin Endocrinol (Oxf). 2007;66(2):251–257. doi:10.1111/j.1365-2265.2006.02717 10.1111/j.1365-2265.2006.02717.x 17223996

[9] Arnaldi G, Tirabassi G, Papa R (2009). Human corticotropin releasing hormone test performance in the differential diagnosis between Cushing’s disease and pseudo-Cushing state is enhanced by combined ACTH and cortisol analysis. Eur J Endocrinol.

[10] Tirabassi G, Papa R, Faloia E, Boscaro M, Arnaldi G (2011). Corticotrophin-releasing hormone and desmopressin tests in the differential diagnosis between Cushing’s disease and pseudo-Cushing state: a comparative study. Clin Endocrinol (Oxf).

[11] Vassiliadi DA, Tsagarakis S (2018). Diagnosis of endocrine diseases: The role of the desmopressin test in the diagnosis and follow-up of Cushing’s syndrome. Eur J Endocrinol.

[12] Moro M, Putignano P, Losa M, Invitti C, Maraschini C, Cavagnini F (2000). The desmopressin test in the differential diagnosis between Cushing’s disease and pseudo-Cushing states. J Clin Endocrinol Metab.

[13] Tirabassi G, Faloia E, Papa R, Furlani G, Boscaro M, Arnaldi G (2010). Use of the desmopressin test in the differential diagnosis of pseudo-Cushing state from Cushing’s disease. J Clin Endocrinol Metab.

[14] Rollin GA, Costenaro F, Gerchman F, Rodrigues TC, Czepielewski MA (2015). Evaluation of the DDAVP test in the diagnosis of Cushing’s Disease. Clin Endocrinol (Oxf).

[15] Araya AV, Romero C, Lemp M (2017). Combined dexamethasone and desmopressin test in the differential diagnosis of ACTH-dependent Cushing’s syndrome and pseudo-cushing’s states. Pituitary.

[16] Martin NM, Dhillo WS, Meeran K (2007). The dexamethasone-suppressed corticotropin-releasing hormone stimulation test and the desmopressin test to distinguish Cushing’s syndrome from pseudo-Cushing’s states. Clin Endocrinol (Oxf).

[17] Fleseriu M, Auchus R, Bancos I (2021). Consensus on diagnosis and management of Cushing’s disease: a guideline update. Lancet Diabetes Endocrinol.

